# Roles of posttranslational modifications in lipid metabolism and cancer progression

**DOI:** 10.1186/s40364-024-00681-y

**Published:** 2024-11-18

**Authors:** Tianyu Feng, He Zhang, Yanjie Zhou, Yalan Zhu, Shiya Shi, Kai Li, Ping Lin, Jie Chen

**Affiliations:** 1 Department of Laboratory Medicine, West China Hospital, Sichuan University, #37, Guo Xue Lane, Chengdu, Sichuan Province 610041 China; 2 Cancer Center and Lab of Experimental Oncology, State Key Laboratory of Biotherapy, and Frontiers Science Center for Disease-related Molecular Network, West China Hospital, Sichuan University, #37, Guo Xue Lane, Chengdu, Sichuan Province 610041 China; 3 Sichuan Clinical Research Center for Laboratory Medicine, Chengdu, Sichuan Province, 610041, China; 4 Clinical Laboratory Medicine Research Center of West China Hospital, #37, Guo Xue Lane, Chengdu, Sichuan Province 610041 China

**Keywords:** Lipid metabolism, PTMs, Cancer progression, PROTAC

## Abstract

Lipid metabolism reprogramming has emerged as a hallmark of malignant tumors. Lipids represent a complex group of biomolecules that not only compose the essential components of biological membranes and act as an energy source, but also function as messengers to integrate various signaling pathways. In tumor cells, de novo lipogenesis plays a crucial role in acquiring lipids to meet the demands of rapid growth. Increasing evidence has suggested that dysregulated lipid metabolism serves as a driver of cancer progression. Posttranslational modifications (PTMs), which occurs in most eukaryotic proteins throughout their lifetimes, affect the activity, abundance, function, localization, and interactions of target proteins. PTMs of crucial molecules are potential intervention sites and are emerging as promising strategies for the cancer treatment. However, there is limited information available regarding the PTMs that occur in cancer lipid metabolism and the potential treatment strategies associated with these PTMs. Herein, we summarize current knowledge of the roles and regulatory mechanisms of PTMs in lipid metabolism. Understanding the roles of PTMs in lipid metabolism in cancer could provide valuable insights into tumorigenesis and progression. Moreover, targeting PTMs in cancer lipid metabolism might represent a promising novel therapeutic strategy.

## Introduction

The reprogramming of metabolic pathways is recognized as a characteristic that supports the survival, proliferation, and dissemination of tumor cells [1, 2]. The glucose, lipid, and amino acid metabolic pathways are three essential nutrient substance metabolic pathways in the human body. The biosynthesis of nucleotides, proteins, and fatty acids (FAs) is upregulated in cancer cells to meet the demands of anabolic growth, leading to extensive metabolic reprogramming. Extensive evidence indicates that dysregulated lipid metabolism is a key metabolic alteration in cancer [3, 4]. Lipids, including sterols, monoglycerides, diacylglycerides, triglycerides, phospholipids, and glycolipids, play crucial roles not only as essential components of biological membranes, second messengers and hormones, but also as important energy sources when glucose availability is limited [3, 5]. In most mammalian tissues, lipid requirements are met through the uptake of free fatty acids (FFAs) and lipoproteins from the bloodstream, with the liver, adipose tissue, and small intestine being the primary sites for fatty acid and cholesterol biosynthesis. Notably, many cancers exhibit a preference for de novo lipogenesis to synthesize most cellular fatty acids, making them less reliant on externally supplied lipids [6]. Thus, de novo lipogenesis is crucial for tumorigenesis and cancer progression. Targeting altered lipid metabolism in cancer cells is increasingly recognized as a hopeful therapeutic strategy for anticancer treatment [7, 8].

Protein posttranslational modifications (PTMs), which are covalent, enzymatic, or non-enzymatic attachments of specific chemical groups to amino acid side chains, increase the functional diversity of the proteome through the covalent addition of functional groups or proteins, proteolytic cleavage of regulatory subunits, or degradation of whole proteins [9]. PTMs affect diverse protein activities and functions, including protein enzymatic activity, subcellular location, protein interactions and stability, and further contribute to multiple cellular biological processes, such as differentiation, gene regulation, signal transduction, cell cycle control and DNA repair [10, 11]. Emerging evidence illustrates that PTMs are widely involved in regulating numerous essential proteins that contribute to the “hallmarks of cancer” [12, 13]. Undoubtedly, the dysregulation of protein PTMs, through which the cellular functions of oncoproteins, tumor suppressors, enzymes, and transcription factors are modulated, is profoundly implicated in the onset and progression of cancers [14, 15]. Protein phosphorylation, methylation, acetylation, SUMOylation, neddylation, ubiquitination, glycosylation, palmitoylation, glutathionylation, S-nitrosylation, and ADP ribosylation are among the most common PTMs [13]. Notably, phosphorylation and acetylation are pivotal modifications extensively studied in metabolic proteins that are intricately linked to the regulation of energy metabolic reprogramming [12, 16]. The cellular energy sensor AMP-activated protein kinase (AMPK) is recognized as negative regulator of cancer lipid synthesis, because of AMPK can directly phosphorylate and inhibit the activities of sterol regulatory element-binding protein (SREBP), acetyl-coA carboxylase (ACC), glycerol-3-phosphate acyltransferase (GPAT), hormone-sensitive lipase (HSL), and HMG-CoA reductase (HMGCR) [17]. The upstream regulators of metabolic enzymes and metabolic pathway fluxes are also regulated by acetylation [18]. For instance, the reversible acetylation of C/EBPα (CCAAT/enhancer-binding protein alpha) can modify its transcriptional function, thereby influencing the expression of metabolic genes and playing a critical role in maintaining cellular metabolic homeostasis [19]. The inhibition of heat shock protein 90 beta (HSP90β) facilitates the ubiquitination and proteasomal degradation of mature sterol regulatory element-binding proteins (SREBPs), leading to the amelioration of lipid metabolism disorders in patients with nonalcoholic fatty liver disease (NAFLD) and diet-induced obese (DIO) mice [20]. The protein stability of ATP-citrate lyase (ACLY) and fatty acid synthase (FASN) is controlled through the dynamic processes of acetylation and deacetylation, thereby regulating de novo lipogenesis and tumor growth [21, 22]. These findings indicates that metabolic enzymes and their upstream regulators are widely regulated by protein PTMs. Zhu et al. focused on the roles of PTMs in rewiring cancer lipid metabolism and highlighted the functions of PTMs in tumor microenvironment [23]. In this review, we highlight a series of studies focusing on the PTMs of key proteins, such as CD36, LDLR, ACLY, FASN, ACC1, SREBPs, and HMGCR, involved in lipid metabolism. We aim to elucidate the PTMs of key enzymes involved in lipid metabolism and their roles in the initiation and progression of cancer and highlight diverse compounds that target either PTMs or upstream regulators in cancer lipid metabolism.

## General overview of lipid metabolism in *cancer* cells

**Fig. 1:**
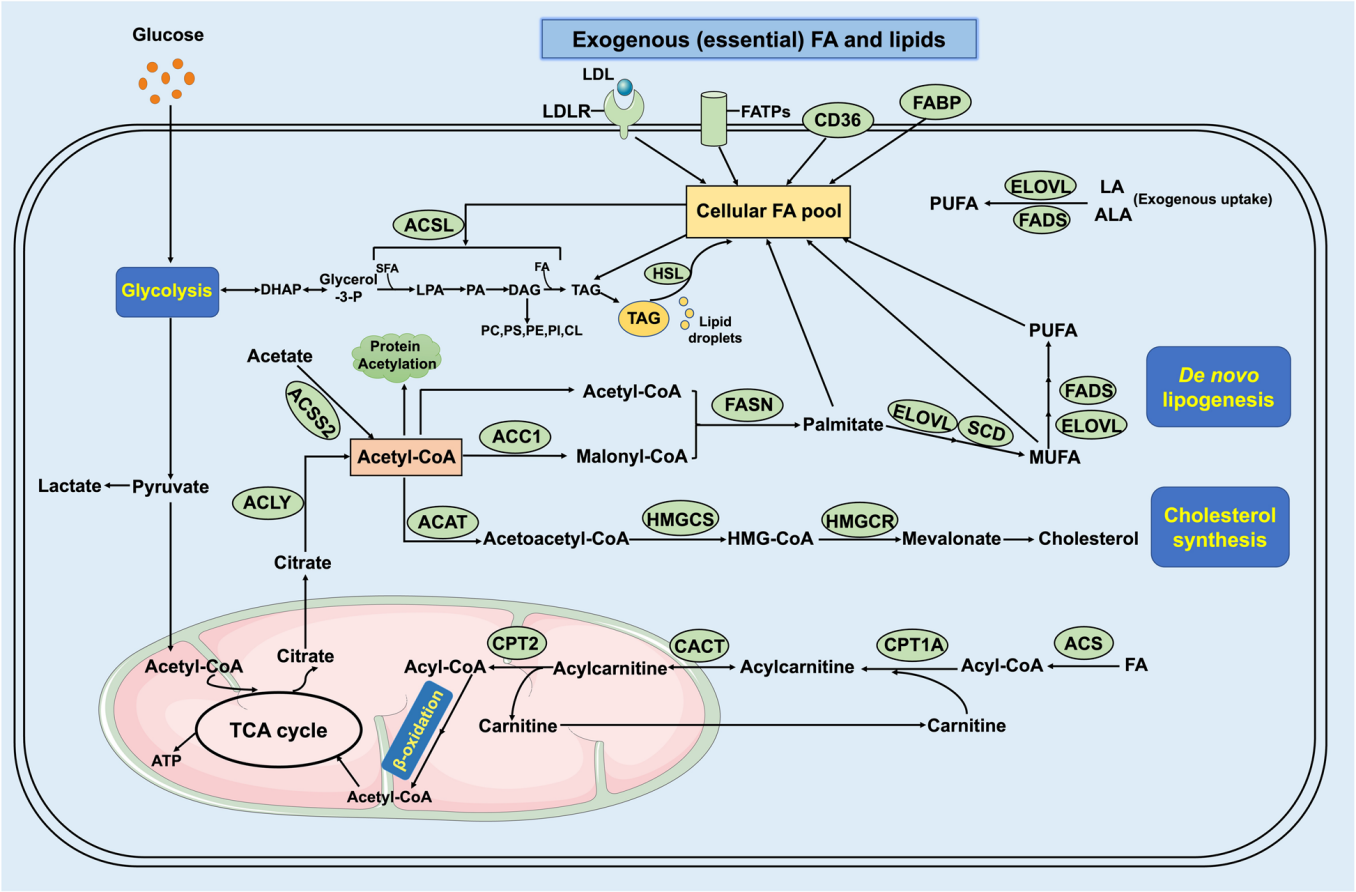
Signaling pathways of lipid metabolism in cancer Glucose uptake and glycolysis generate pyruvate and acetyl-CoA to support the TCA cycle. FAO is another source of acetyl-CoA in the mitochondria for TCA cycle. Cytoplasmic acetyl-CoA is synthesized from citrate catalyzed by ACLY and acetate catalyzed by ACSS2. Cytoplasmic acetyl-CoA is then converted into palmitate by a series of biosynthetic reactions catalyzed by ACC1 and FASN. Palmitate is further modified by ELOVL enzymes to generate SFA. MUFA is produced by desaturation of SFA catalyzed by SCD1or FADS2. Moreover, LA and ALA from exogenous uptake can generate different PUFA through FADS and ELOVL enzymes. Furthermore, acetyl-CoA also supports the mevalonate pathway to produce cholesterol catalyzed by ACAT, HMGCS and HMGCR. Exogenous (essential) FAs and lipids are taken up from the bloodstream through LDLR, FATPs or CD36 together with FABPs. Collectively, FAs from exogenous uptake and generation form de novo synthesis make up the cellular FA pool. ACAT, acetyl-CoA acetyltransferase; ACLY, ATP-citrate lyase; ACSS2, acetyl-CoA synthetase 2; ACC1, acetyl-CoA carboxylases 1; ALA, α-linolenic acid; ELOVL, elongation of very long lipids protein; FABPs, FA binding proteins; FAO, fatty acid oxidation; FADS2, fatty acid desaturase 2; FASN, fatty acid synthase; FATPs, FA transport proteins; HMGCS, HMG-CoA synthetase; HMGCR, HMG-CoA reductase; LA, linoleic acid; LDLR, low-density lipoprotein (LDL) receptor; MUFA, monounsaturated fatty acids; PUFA, poly-unsaturated fatty acid; SFA, saturated fatty acid; SCD1, stearoyl-CoA desaturase 1; TCA, tricarboxylic acid

Lipid metabolism can be divided into two processes: lipids acquisition and lipids utilization (Fig. 1). In terms of acquisition, mammalian cells obtain lipids by either taking them from an external source (such as FFAs or lipoproteins) or synthesizing them through de novo fatty acid synthesis. Exogenous free fatty acids presented in the local microenvironment can be transported into the cell via the fatty acid translocase (FAT, also known as CD36) or the fatty acid transport proteins (FATPs) [24]. FA binding proteins (FABPs) also participate in the uptake and transport of FAs. Moreover, LDL- and VLDL-derived lipids are alternative ways through which cancer cells can acquire essential fatty acids. In cancer cells, FA uptake, de novo lipogenesis and FA oxidation (FAO, also known as β-oxidation) are elevated to produce abundant energy and to accumulate lipids.

A wide variety of cancers are well-known to undergo exacerbated de novo synthesis irrespective of the levels of circulating lipids [25]. The de novo fatty acid pathway begins via the conversion of citrate to acetyl-coenzyme A (CoA) and oxaloacetate. This process is catalyzed by ACLY. Subsequently, ACC1 converts acetyl-CoA to malonyl-CoA, marking the first committed and energy-consuming step in de novo fatty acid synthesis. FASN is then responsible for producing basic 16-carbon saturated fatty acids, such as palmitic acid, using acetyl-CoA, malonyl-CoA, and NADPH. The family of elongation of very-long-chain fatty acids (ELOVL), the first rate-limiting enzymes in the synthesis of long-chain fatty acids, oversees the elongation and desaturation of newly synthesized fatty acids to produce long-chain fatty acids such as stearic acid. In the production of monounsaturated fatty acids (MUFAs), stearoyl-CoA desaturase 1 (SCD1) acts as a rate-limiting enzymes. Subsequently, MUFAs serve as the primary substrates for synthesizing various lipid types, such as phospholipids, triglycerides, wax esters, and cholesteryl esters. Moreover, membrane-bound transcription factors SREBPs play a central role in lipid metabolism by controlling the synthesis of fatty acids, triglycerides, and cholesterol [26, 27]. SREBP-1 governs ACLY, ACACA, and FASN in the fatty acid biosynthesis pathway, whereas SREBP-2 regulates HMGCS (3-hydroxy-3-methylglutaryl-CoA synthase 1) and HMGCR (3-hydroxy-3-methylglutaryl-CoA reductase) in cholesterol synthesis [28, 29]. For the synthesis of triacylglycerol (TAG), fatty acids need to be converted into acyl-CoA by acyl-CoA synthetase (ACS). Subsequently, acyl-CoA is transformed into TAG through a series of enzymatic reactions involving glycerol-3-phosphate acyltransferase (GPAT), acylglycerolphosphate acyltransferase (AGPAT), phosphatidic acid phosphohydrolase (PAP or lipin), and diacylglycerol acyltransferase (DGAT).

TAG stored in lipid droplets as an energy source can be mobilized through lipolysis catalyzed by lipases, resulting in FA release [30]. FAs are catabolized via the FAO pathway [30, 31]. Many kinds of cancer cells rely on FAO for proliferation, survival, stemness, drug resistance, and metastatic progression [31]. For instance, carnitine palmitoyltransferase 1 (CPT1), the rate-setting enzyme in FAO, is responsible for the growth and/or viability of myeloid leukemia [32, 33], ovarian cancer [34], hepatocellular carcinoma [35], prostate cancer [36] and glioblastoma cells [37]. Fatty acids are first activated into fatty acyl-CoA by ACS to undergo FAO. Fatty acyl-CoA then conjugates with carnitine and is converted into acylcarnitine by CPT1, which is located on the outer mitochondrial membrane. Acylcarnitine is subsequently translocated into the mitochondria through the carnitine-acylcarnitine translocase (CACT) and reconverted to acyl-CoA by carnitine palmitoyltransferase 2 (CPT2) on the inner mitochondrial membrane. Within the mitochondrion, acyl-CoA undergoes a series of enzymatic reactions in a repeated four-step cycle to be cleaved into acetyl-CoA. Acetyl-CoA then enters the tricarboxylic acid (TCA) cycle, which is coupled to oxidative phosphorylation to generate ATP.

## PTMs of key molecules in lipid metabolism

**Fig. 2:**
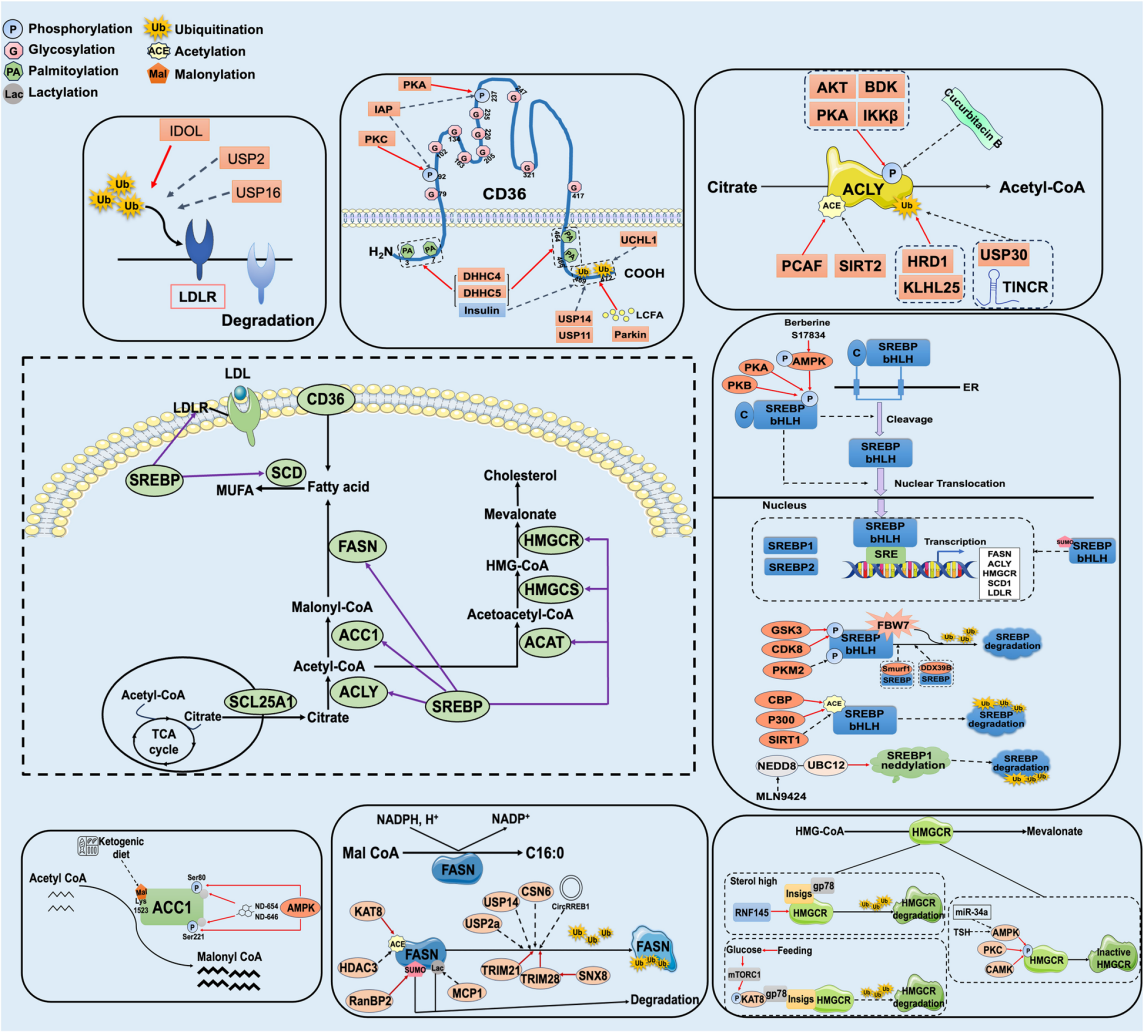
Posttranslational modification control of lipid metabolism-related enzymes The red solid arrow represents positive regulation, the black dashed arrow indicates negative regulation, and the purple solid arrow shows transcriptional regulation. Enzymes that are transcriptional regulated by SREBPs are shown in red box. For LDLR, IDOL promotes the ubiquitylation and degradation of LDLR protein, while USP2 and USP16 inhibit LDLR ubiquitination. Additionally, CD36 is phosphorylated by PKA, PKC and dephosphorylated by IAP. Palmitoyl transferases DHHC4/5 promote palmitoylation of CD36. The extracellular loop of CD36 has 10 N-linked glycosylation sites, which are mediated by glycosyltransferase. E3 ligase Parkin promotes the monoubiquitylation of CD36 and enhances its stability. LCFAs promote polyubiquitination and degradation of CD36. Oppositely, insulin inhibits ubiquitination of CD36, deubiquitinating enzymes UCHL1, USP14 and USP11 also prevent the proteasome degradation of CD36. Besides, ACLY is affected by protein phosphorylation, acetylation and ubiquitylation. AKT, BDK, PKA and IKKβ phosphorylate ACLY. Acetyltransferase PCAF modulates acetylation of ACLY, while SIRT2 regulates its deacetylation. KLHL25 and HRD1 facilitate the ubiquitination of ACLY, while USP30 modulates its deubiquitylation. Moreover, phosphorylation of precursor forms of SREBPs is mediated by AMPK, PKA and PKB, which inhibits the cleavage and nuclear translocation of SREBPs. SUMOylation suppresses the transcriptional activities of SREBPs. Furthermore, GSK-3 and CDK8 phosphorylate active nuclear fragments of SREBPs and further create binding sites for the FBXW7, resulting in ubiquitination and degradation of SREBPs. Acetylation of SREBPs impedes their ubiquitination and inhibits protein degradation. The neddylation of SREBP-1 by UBE2M increases its stability by reducing ubiquitination. AMPK phosphorylates ACC1 and suppresses its enzymatic activity. FASN are also regulated by PTMs. TRIM21 and TRIM28 promote the ubiquitin‒proteasome pathway mediated degradation of FASN, while USP2a and USP14 inhibit its protein degradation. Acetylation, SUMOylation and lactylation of FASN also contribute to its ubiquitination and degradation. When sterol is high, HMGCR is rapidly degraded through RNF145 and gp78-mediated ubiquitination. HMGCR can also be phosphorylated by AMPK, PKC and CAMK and attenuates its activity. ACLY, ATP-citrate lyase; ACSS2, acetyl-CoA synthetase 2; ACC1, acetyl-CoA carboxylases 1; AKT, serine/threonine kinase 1; AMPK, AMP-activated protein kinase; BDK, Branched-chain alpha-keto acid dehydrogenase kinase; CAMK, Calcium/calmodulin-dependent protein kinases DHHC4/5, zinc finger DHHC-type palmitoyltransferase 4/5; FASN, fatty acid synthase; FATPs, FA transport proteins; FBXW7, F-box and WD repeat domain containing 7; HMGCR, HMG-CoA reductase; HRD1, HMG-CoA reductase degradation protein; IDOL, inducible degrader of the low-density lipoprotein receptor; IAP, intestinal alkaline phosphatase; KLHL25, Kelch-like family member 25; LDLR, low-density lipoprotein (LDL) receptor; LCFAs, long-chain fatty acids; PKA, protein kinase A; PCAF, P300/CBP-associated factor; PKC, protein kinase C; SCD1, stearoyl-CoA desaturase 1; SREBPs, Sterol regulatory element-binding protein; SIRT2, Sirtuin; UBE2M, Ubiquitin conjugating enzyme E2 M; USP, Ubiquitin-specific protease; UCHL1, ubiquitin C-terminal hydrolase L1

PTMs play a crucial role in regulating lipid metabolism, especially in the process of de novo lipogenesis. Of noted, lipid metabolism-related key proteins and rate-limiting enzymes are widely regulated by PTMs (Table 1 and Fig. 2). The PTMs of pivotal proteins during the process of lipid metabolism are described as below.

**Table 1:** Posttranslational modification control of lipid metabolism enzymes

Molecule	Regulator	Modification and sites	Biological functions	Effects on cancer	Refs
**CD36**	PKA/PKC	Phosphorylation, Ser237	Deactivates CD36		[38]
	LCAF	Ubiquitylation, Lys469, Lys472	Degrades CD36		[39]
	Insulin	Inhibition of ubiquitylation	Stabilizes CD36		[39]
	Parkin	Monoubiquitylation	Stabilizes CD36		[40]
	USP14	Inhibition of ubiquitylation	Stabilizes CD36		[41]
	FA/HFD	O-GlcNAcylation, Ser468, Thr470	Activates CD36	Promotes the metastasis of gastric cancer cells in vivo and in vitro	[42]
	Insulin	Palmitoylation	Activates CD36		[43]
	DHHC4/DHHC5	Palmitoylation	Stabilizes CD36		[44]
	Palmitic acid (PA)	Palmitoylation	Stabilizes CD36	Protects cancer cells against palmitate-induced toxicity	[45]
**LDLR**	IDOL	Ubiquitylation	Degrades LDLR		[46]
	USP2/USP16	Inhibition of ubiquitylation	Stabilizes LDLR	Promotes the uptake of LDL in human cervical tumor cell lines	[47]
**ACLY**	PI3K-AKT	Phosphorylation, Ser454	Activates and stabilizes ACLY	Correlates with tumor differentiation and poor prognosis of non–small cell lung cancer	[48, 49]
	AKT-mTORC2/ AKT-ATM	Phosphorylation, Ser455	Activate ACLY		[50]
	BDK	Phosphorylation, Ser454	Activates ACLY		[51]
	PCAF	Acetylation, Lys540, 546, 554	Stabilizes ACLY	Enhances de novo lipid synthesis and promotes cell proliferation of lung carcinoma	[21]
	SIRT2	Deacetylation	Destabilizes ACLY		[21]
	UBR4/ CUL3-KLHL25	Ubiquitylation, Lys540, 546, 554	Degrade ACLY	Inhibits the lipid synthesis, cell proliferation, and tumor growth of human lung cancer	[52]
	HRD1	Ubiquitylation	Degrades ACLY		[53]
	LncRNA TINCR	Inhibition of ubiquitylation	Stabilizes ACLY	Promotes lipid biosynthesis and the progression of nasopharyngeal carcinoma	[54]
	USP30	Deubiquitylation	Stabilizes ACLY	Promotes lipogenesis and tumorigenesis of HCC	[55]
**ACC1**	AMPK	Phosphorylation, Ser80	Inhibits ACC1		[56]
	ND-654/ND-646	Mimics Phosphorylation	Inhibits ACC1	Suppresses de novo fatty acid synthesis and tumor growth	[56]
	Ketogenic diet	Malonylation, Lys1523	Activates ACC1		[57]
**ACC2**	AMPK	Phosphorylation, Ser221	Inhibits ACC2		[56]
**FASN**	KAT8	Acetylation	Degrades FASN	Inhibits de novo lipogenesis and suppresses tumor growth	[22]
	HDAC3	Deacetylation	Stabilizes FASN		[22]
	USP2a	Deubiquitylation	Stabilizes FASN	Enhances tumor progression of bladder cancer	[58, 59]
	USP14	Deubiquitylation	Stabilizes FASN	Promotes proliferation of human prostate cancer cell line	[60, 61]
	TRIM21	Ubiquitylation	Degrade FASN	Inhibits lipogenesis of HCC cells and hepatocarcinogenesis in DEN/CCL_4_ mice model	[22, 62]
	CSN6	Inhibition of ubiquitylation	Stabilizes FASN	Enhances lipid contents and promotes colorectal cancer growth	[63]
	GSK3β	Phosphorylation	Destabilizes FASN	Decreases lipid contents and inhibits colorectal cancer growth	[63]
	CircRREB1	Inhibition of ubiquitylation	Stabilizes FASN		[64]
	SNX8	Ubiquitylation	Degrades FASN		[65]
	RanBP2	SUMOylation	Stabilizes FASN		[64]
	MPC1	Lactylation, Lys673	Degrades FASN		[66]
**SREBP1a**	P300	Acetylation, Lys333, Lys324	Stabilize SREBP1a		[67]
	FBXW7	Ubiquitylation	Degrade SREBP1a	Suppresses de novo lipogenesis and tumor growth	[68, 69]
	UBC9	SUMOylation, Lys123, Lys418	Deactivates SREBP1a		[70]
	UBC12	Neddylation	Stabilize SREBP1a	Promotes the metabasis of HCC and breast cancer cells	[71]
	GSK-3	Phosphorylation, Thr426, Ser430, Ser434	Degrade SREBP1a		[68, 72]
	ERK1/2	Phosphorylation, Ser117	Activates SREBP1a		[73, 74]
	JNK1/2	Phosphorylation, Ser117	Activates SREBP1a		[73, 74]
	P38-MAPK	Phosphorylation, Ser63, Thr426	Activates SREBP1a		[74]
	PKM2	Phosphorylation, Thr-59	Stabilize nuclear SREBP1a	Activates lipogenesis and HCC cell proliferation	[75]
**SREBP1c**	PKA	Phosphorylation, Ser338	Inhibits SREBP1a		[76]
	P300	Acetylation, Lys289, Lys309	Stabilizes SREBP1c		[72]
	SIRT1	Deacetylation	Destabilizes SREBP1c		[72]
	FBXW7	Ubiquitylation	Degrades SREBP1c	Suppresses de novo lipogenesis and tumor growth	[68]
	Smurf1	Inhibition of ubiquitylation	Stabilizes SREBP1c		[77]
	P38	Phosphorylation, Ser39, Thr402	Activates SREBP1c		[78]
	ERK-MAPK	Phosphorylation, Thr81, Ser93	Activates SREBP1c		[73, 74]
	JNK-MAPK	Phosphorylation, Thr81, Ser93	Activates SREBP1c		[78]
	AMPK	Phosphorylation, Ser372	Inhibits and deactivates SREBP1c		[79]
	Berberine	Phosphorylation, Ser372	Inhibits and deactivates SREBP1c	Reduces lipogenesis and suppresses colon cancer cell proliferation	[80, 81]
**SREBP2**	CPB/P300	Acetylation	Activates SREBP2		[68]
	FBXW7	Ubiquitylation	Degrades SREBP2		[70]
	/	SUMOylation, Lys464	Degrades SREBP2		[70]
	ERK-MAPK	Phosphorylation, Ser432, Ser455	Activates SREBP2		[73, 74]
	AMPK	Phosphorylation	Deactivates SREBP2		[73, 74, 79]
**HMGCR**	TSH	Inhibition of phosphorylation	Activates HMGCR		[82]
	miR-34a	Inhibition of phosphorylation	Activates HMGCR		[83]
	RNF145	Ubiquitylation	Degrades HMGCR		[84]
	USP20	Deubiquitylation	Stabilizes HMGCR		[85]

### Phosphorylation, ubiquitination, glycosylation, and palmitoylation of CD36

CD36 is a transmembrane glycoprotein and facilitates the cellular uptake of long-chain fatty acids (LCFAs) [86]. CD36 is highly expressed in many types of cancers, including HCC, breast cancer, oral squamous cell carcinoma, acute myeloid leukemia and CRC [87–89]. CD36 plays important role in cancer progression by promoting the stemness, growth and metastasis of cancer cells [90]. The protein synthesis, distribution, and function of CD36 are widely regulated by PTMs, including phosphorylation, ubiquitination, glycosylation, and palmitoylation [91]. CD36 is ubiquitinated at Lys 469 and Lys 472 on its C-terminal domain, which is increased by LCFAs but decreased by insulin [39]. CD36 is monoubiquitinated and stabilized by the E3 ligase Parkin [40, 92]. Ubiquitin-specific protease 14 (USP14) also modulates the protein stability of CD36 by cleaving ubiquitin chains from ubiquitinated CD36 and preventing its degradation via proteasome, whereas inhibition of USP14 suppressed foam cell formation by impairing lipid uptake [41]. The glycosylation of CD36 occurs on asparagine residues and is mediated by glycosyltransferase. The glycosylation of CD36 could enhance the stability of the tertiary folding of polypeptides and therefore influence the translocation of CD36 to the cell membrane. FA-induced O-GlcNAcylation of CD36 at Ser468 and Thr470, leading to enhanced FA uptake activity of CD36, which drives gastric cancer metastasis [42]. Palmitoylation, a vital posttranslational modification of the CD36 protein catalyzed by palmitoyl acyltransferases (PATs), plays a crucial role in its function [44]. Stimulation by insulin boosts the palmitoylation of CD36, while mutations in palmitoylation sites impede CD36 translocation to the cell membrane [43]. Elevated palmitoylation of CD36 promotes the uptake of fatty acids, thereby accelerating liver steatosis and fibrosis [43]. A newly study demonstrates that CD36 palmitoylation promotes MUFA-specific uptake and protects cancer cells from palmitate-induced toxicity [45]. In addition, Thr92 and Ser237 of CD36 can be phosphorylated by protein kinase C (PKC) and protein kinase A (PKA), respectively [93]. The two phosphorylation sites of CD36 are essential for adhesion capacity and platelet reactivity in resting platelets and a megakaryocytic cell line [38]. However, studies of CD36 phosphorylation in tumor cells are insufficient. However, additional research is needed to explore the significance of CD36 phosphorylation in cancer.

### Ubiquitylation of LDLR

The low-density lipoprotein receptor (LDLR), the major cholesterol-carrying lipoprotein of plasma, is normally bound at the cell membrane, where it binds low-density lipoprotein/cholesterol to take them into the cell. LDLR is also upregulated in CRC, lung cancer, breast cancer and pancreatic cancer [94–97]. The gene transcription of *LDLR* is primarily regulated by SREBP2 [98]. In addition to transcriptional regulation, posttranscriptional degradation is also a vital determinant of LDLR abundance and LDLR-mediated lipoprotein metabolism [47, 99, 100]. Importantly, reversible ubiquitylation and deubiquitylation of LDLR play key roles in controlling intracellular trafficking of the receptor. The ubiquitination and degradation of LDLR is regulated by E3 ubiquitin ligase inducible degrader of the low-density lipoprotein receptor (IDOL) [99]. Liver X receptor (LXR), a sterol-sensitive nuclear receptor, inhibits LDLR-dependent cholesterol uptake independent of SREBPs by inducing IDOL-mediated ubiquitination and degradation of LDLR [99]. Mechanistically, the IDOL FERM domain binds directly to a recognition sequence in the cytoplasmic tails of lipoprotein receptors [46]. The USP family of deubiquitylates was identified as a negative regulator of IDOL-dependent degradation of LDLR. Ubiquitin-specific protease 2 (USP2) counteracts IDOL-mediated degradation of LDLR, resulting in the increased LDLR abundance and LDL uptake [100]. A recent study demonstrated that ubiquitin-specific protease 16 (USP16) could inhibit LDLR ubiquitination and degradation, promoting the uptake of LDL in human cervical tumor cell lines [47].

### Acetylation, ubiquitylation and phosphorylation of ACLY

Acetyl-CoA acts as a critical intermediate in glucose, lipid, and amino acid metabolism, serving as a precursor for the fatty acid and mevalonate synthesis pathways. ACLY, a cytosolic enzyme, plays a key role in converting citrate derived from the mitochondria into acetyl-CoA, thus bridging the gap between glucose and/or glutamine metabolism and fatty acid synthesis and/or the mevalonate pathway [101]. The upregulated expression and activity of ACLY have been reported in lung, prostate, bladder, breast, liver, colon, and gastric cancer, whereas the inhibition of ACLY suppresses the proliferation of tumor cells [48, 102–105]. The stability of ACLY is intricately controlled by protein acetylation and ubiquitylation. Specifically, acetylation at three lysine residues (Lys540, Lys546, and Lys554) prevents the ubiquitylation and subsequent degradation of ACLY. P300 and calcium-binding protein (CBP)-associated factor (PCAF) are the acetyltransferase of ACLY, whereas ACLY deacetylation is modulated by Sirtuin 2 (SIRT2). Remarkably, the acetylation of ACLY results in its accumulation and enhanced fatty acid synthesis, thereby promoting tumor growth in lung carcinoma [21]. The ubiquitination and subsequent degradation of ACLY are directly mediated by HMG-CoA reductase degradation protein (HRD1), which contributes to the mitigation of NAFLD in genetically diabetic leptin receptor-mutated (db/db) mice [53]. In lung cancer cells, ACLY is specifically targeted for degradation by the Cullin3–KLHL25 ubiquitin ligase system [52]. The ubiquitination of ACLY is also modulated by long noncoding RNAs (lncRNAs). In nasopharyngeal carcinoma cells, the lncRNA TINCR interacts directly with ACLY, thereby preserving ACLY protein stability by impeding its ubiquitination-mediated degradation [54]. The deubiquitination of ACLY is facilitated by USP30 [55]. In mice, the knockout of USP30 considerably reduces DEN-induced hepatocarcinogenesis and inflammation [55]. Moreover, ACLY can be activated through phosphorylation. In vitro, recombinantly expressed human ACLY was phosphorylated either by PKA alone or in combination with GSK-3, and the enzyme activity of rACLY increases [106]. Ser454 of ACLY is a direct substrate for its phosphorylation by active serine/threonine kinase 1 (AKT) in normal rat adipocytes [49]. In brown adipocytes, Ser455 of ACLY undergoes phosphorylation in a mechanistic/mammalian target of rapamycin complex 2 (mTORC2)/AKT-dependent manner. This phosphorylation event serves to enhance acetyl-CoA synthesis and facilitates de novo lipogenesis following glucose uptake and glycolysis [50]. In Wistar rats, ACLY is phosphorylated at Ser454 by branched-chain alpha-keto acid dehydrogenase kinase (BDK) in an AKT-independent manner, leading to increased de novo lipogenesis [51]. In human lung adenocarcinoma, phosphorylated ACLY, which is directly regulated by the phosphoinositide 3-kinase (PI3K)-AKT pathway, contributes to ACLY protein stabilization [48]. Consequently, p-ACLY levels are elevated in lung adenocarcinomas and associated with the aggressive biological behavior of these tumors [48]. Given its diverse and dynamic posttranslational modifications, ACLY is a promising candidate for cancer treatment.

### Phosphorylation of ACC1 and ACC2 and malonylation of ACC1

Acetyl-CoA carboxylases (ACCs), which include ACC1 and ACC2, are enzymes responsible for catalyzing the carboxylation of acetyl-CoA to produce malonyl-CoA [107]. ACC1 plays a crucial role in catalyzing this conversion, serving as the rate-limiting step in de novo fatty acid synthesis, whereas ACC2 is involved in regulating FAO. The identification of numerous phosphorylation sites in mammalian ACC1 and ACC2 highlights ACC as a hopeful target for drug development. Compelling evidence has confirmed that in humans, AMPK, a serine/threonine kinase, directly suppresses the enzymatic activities of ACC1 and ACC2 by phosphorylating Ser80 and Ser221, respectively [56]. ND-654, a novel liver-specific ACC1 inhibitor that mimics the effects of ACC phosphorylation, has been demonstrated to suppress hepatic de novo lipogenesis and HCC progression [56]. The introduction of experimental ACC mutants lacking AMPK phosphorylation sites (ACC1_Ser79Ala and ACC2_Ser212Ala) has been found to protect against the growth inhibition caused by cetuximab in head and neck squamous cell carcinoma [108]. ND-646 is a small molecule allosteric inhibitor of ACC1 and ACC2 and interacts with the residues located within the dimerization site of the biotin carboxylase domain of ACC required for AMPK binding. In non-small cell lung cancer (NSCLC), ND-646 effectively inhibits de novo fatty acid synthesis, leading to the remarkable suppression of tumor growth in vitro and in vivo [108]. Intriguingly, a recent study has revealed that the malonylation of ACC1 at Lys1523 enhances its activity and stability. Furthermore, in NAFLD, the lysine malonylation of ACC1 is reduced by a ketogenic diet, thereby accelerating hepatic steatosis [57]. However, the specific modification site, function, and regulation of ACC1 malonylation in cancer cells warrant further investigation.

### Acetylation, ubiquitylation, SUMOylation and lactylation of FASN

FASN, a well-studied de novo lipogenesis enzyme in cancer, plays a pivotal role in the synthesis of palmitate (C16:0) from acetyl-CoA and malonyl-CoA. FASN is overexpressed in various cancers and is vital for the enhanced production of FAs. FASN is tightly regulated by PTMs, making it as an appealing target for cancer therapy. Isopeptidase USP2a deubiquitinates FASN, shielding it from proteasome-mediated degradation and promoting survival in prostate cancer [58]. Furthermore, FASN is a substrate of USP14 that directly interacts with and enhances FASN stability. USP14 overexpression has been shown to accelerate hepatosteatosis in C57BL/6 mice [60]. The E3 ubiquitin ligase TRIM21 disrupts FASN stability [22, 62]. The acetylation of FASN by lysine acetyltransferase 8 (KAT8) triggers FASN degradation through the ubiquitin‒proteasome pathway mediated by the E3 ubiquitin ligase TRIM21, leading to a reduction in de novo lipogenesis and tumor cell growth, whereas HDAC3 catalyzes FASN deacetylation [22]. A recent study has demonstrated that in colorectal cancer, COP9 signalosome subunit 6 (CSN6) promotes FASN protein stability by counteracting the activity of E3 ligase F-box and WD repeat domain containing 7 β (FBXW7β). Additionally, the phosphorylation of FASN by glycogen synthase kinase-3β (GSK3β) enhances FBXW7β-mediated FASN ubiquitination and degradation [63]. CircRREB1 contributes to FASN protein stability by interfering with its proteasome-mediated degradation in chondrocytes [64]. Sorting nexin 8 (SNX8) has emerged as a potential therapeutic target for NAFLD because it directly binds to FASN and boosts FASN degradation by facilitating the tripartite motif-containing 28 (TRIM28)-FASN interaction [65]. SUMOylation has also been shown to prevent proteasome degradation and maintain FASN function in breast cancer cells [109]. Moreover, the E3 SUMO protein ligase RanBP2 assists the SUMOylation and stability of FASN, thereby preserving its role in the lipid metabolism of chondrocytes [64]. Lysine lactylation, a newly identified lactate-derived PTM of lysine residues, affects tumor biology through transcriptional regulation and nonhistone protein modulation, providing a promising and potential therapy for target tumors [110, 111]. A recent study has demonstrated that in a NAFLD model, lactylation at the Lys673 site of FASN inhibits fatty acid synthase activity and the knockout of MPC1 promotes FASN lactylation and ultimately improves liver lipid deposition [66]. These intriguing discoveries highlight the potential of targeting FASN PTMs as a therapeutic strategy in cancer.

### Phosphorylation, ubiquitylation, SUMOylation, acetylation and neddylation of SREBPs

SREBPs, transcription factors that orchestrate the transcription of key lipogenic enzymes such as ACC, ACLY, FASN, and SCD, are central players in the biosynthesis of fatty acids and cholesterol within cells [27]. SREBP1a, SREBP1c and SREBP2 are three closely related isoforms identified in mammalian cells and are encoded by the *SREBF1* and *SREBF2* genes [26, 29]. SREBP1 is involved in regulating the synthesis of fatty acids, phospholipids, and triacylglycerols, whereas SREBP2 is primarily responsible for controlling cholesterol biosynthesis. Intriguingly, SREBP1 and SREBP2 oversee a set of genes that are shared between the two pathways, underscoring the interconnected nature of their functions in cellular lipid metabolism [29]. SREBPs are initially synthesized as inactive precursors attached to the endoplasmic reticulum membrane. Through cleavage by site 1 and 2 proteases, the active N-terminal fragments of SREBPs, known as mature or nuclear SREBPs, are released and transported into the nucleus. Once inside the nucleus, they bind to the promoters of the target genes crucial for lipid synthesis and uptake [112]. A growing body of evidence suggests that SREBPs drive the metabolic reprogramming of various cancers and are considered as prognosis biomarkers and potential therapeutic targets in patients with cancer [113]. In addition to proteolytic processing, SREBP1 and SREBP2 undergo several posttranslational modifications, including phosphorylation, acetylation, SUMOylation, ubiquitylation, and neddylation [67, 68, 70–72, 77, 78]. These modifications can influence the activity, stability, and localization of SREBPs, further fine-tuning their regulatory functions in cellular lipid metabolism and potentially affecting cancer progression.

The phosphorylation of both the precursor and mature forms of SREBPs is crucial for the proteolytic maturation and turnover of this essential regulator, ultimately activating de novo lipogenesis in cancer cells. Serine 117 has been identified as a phosphorylation site of SREBP-1a, which is activated by extracellular signal-regulated kinase 1/2 (ERK1/2) and c-Jun N-terminal kinases (JNK) [73, 74]. The Ser93 and Thr81 residues of SREBP-1c are the substrate of JNK. Additionally, p38 MAP kinases can phosphorylate SREBP-1a at Ser63 and Thr426 as well as SREBP-1c at Ser39 and Thr402 [74]. Notably, the inhibition of SREBP-1c phosphorylation leads to a decrease in the de novo lipid synthesis and the expression of SREBP-1c target genes in HepG2 cells exposed to high glucose and lipid levels [78]. Importantly, AMPK-mediated phosphorylation disrupts the nuclear translocation and transcriptional activities of SREBP1c and SREBP2, consequently suppressing hepatocyte lipogenesis [79]. Remarkably, the synthetic polyphenol S17834, known for its potent and sustained activation of AMPK kinase activity, has been demonstrated to repress the cleavage of SREBP-1 [79]. This inhibition, in turn, provides protection against hepatic steatosis, hyperlipidemia, and accelerated atherosclerosis in diet-induced insulin-resistant LDL receptor-deficient mice [79]. Berberine (BBR), a natural compound derived from Chinese herbs, enhances the phosphorylation of AMPK and SREBP-1c at Ser372 in HepG2 cells and the livers of HFD-fed mice, leading to a reduction in liver triglyceride synthesis and the prevention of hepatic steatosis [80]. The cAMP-dependent kinase PKA phosphorylates Ser314 of SREBP-1c and Ser338 of SREBP-1a, resulting in the inhibition of SREBP-1 transactivation and SREBP-1-mediated lipogenesis in HepG2 cells [76]. Furthermore, in rat hepatocytes, PKB directly phosphorylates full-length SREBP-1c, a crucial step for the enhanced interaction between SREBP-1c and COPII proteins triggered by insulin [114].

The active nuclear fragments of SREBPs are widely recognized to be inherently unstable and are subject to degradation through the ubiquitination-dependent proteasome system [115]. FBXW7 has been extensively studied for its pivotal role in modulating the protein stability of the SREBP family in a phosphorylation-dependent manner. Specifically, GSK-3 phosphorylates SREBP1a at Thr426, Ser430, and Ser434, creating binding sites for FBXW7 [68, 72]. As a result, FBXW7 enables the ubiquitination and degradation of nuclear SREBP1c and SREBP2, establishing itself as a crucial regulator of lipid metabolism [68]. Phosphorylation at Thr59 of SREBP1a has been shown to enhance the protein stability of nuclear SREBP-1a and facilitate its interaction with PKM2, thereby stimulating the growth of HepG2 cells [75]. Moreover, cyclin-dependent kinase 8 (CDK8) has been found to phosphorylate the Thr402 site of SREBP-1c directly, triggering the ubiquitination and rapid degradation of SREBP-1c [116]. By contrast, SMURF1 interacts with SREBP-1c, protecting it from ubiquitination and degradation by impeding the interaction of SREBP-1c with FBXW7A [77]. A recent study has revealed that DExD-box helicase 39B (DDX39B) promotes the stabilization of the SREBP1 protein by inhibiting the FBXW7-mediated ubiquitination and degradation of SREBP1, suggesting that DDX39B is a novel therapeutic target in HCC [117].

SUMO modification serves as an alternative mechanism for the negative regulation of SREBPs, distinct from proteolysis-mediated regulation. It has been demonstrated to suppress the transcriptional activity of nuclear SREBP1a and SREBP-2 [70]. Moreover, the phosphorylation of SREBP2 induced by growth factors inhibits SUMOylation, leading to increased lipid uptake and synthesis, which are essential for the proliferation of HepG2 cells [118].

SREBPs are also subject to regulation by protein acetylation. Specifically, the p300-mediated acetylation of SREBP1a at Lys324 and Lys333 and of SREBP-1c at Lys289 and Lys309 impede the ubiquitination of these SREBPs [67]. For SREBP-1c, p300 targets Lys289 and Lys309 for acetylation, whereas SIRT1 is responsible for their deacetylation, as evidenced by recent findings [72]. The deacetylation of SREBP-1c by SIRT1 decreases its activity by destabilizing the protein and disrupting its interaction with the promoters of lipogenic target genes [72]. Notably, a recent study has revealed that mitochondrial fission elevates the acetylation level of SREBP1 by suppressing the NAD^+^/SIRT1 signaling pathway, thereby augmenting SREBP1-mediated de novo lipogenesis in HCC cells [119].

Neddylation, a recently discovered posttranslational modification process, involves the attachment of NEDD8, an ubiquitin-like peptide, to a target substrate protein to modulate its activity or function. A groundbreaking study identifies that SREBP-1 is a novel neddylation substrate of ubiquitin conjugating enzyme E2 M (UBE2M), a NEDD8-conjugating enzyme. The neddylation of SREBP-1 by UBE2M has been found to enhance the stability of SREBP-1 by reducing its ubiquitination [71]. Intriguingly, the use of MLN4924, a specific neddylation inhibitor that forms a stable MLN4924-NEDD8 adduct by binding to NEDD8, has been shown to decrease lipid accumulation by suppressing SREBP1 neddylation in liver cancer cells [71]. However, the specific neddylation sites of SREBPs and the implications of SREBP neddylation in malignant tumors remain unclear.

### Ubiquitylation and phosphorylation of HMGCR

Cholesterol and its metabolites play a critical role in oncogenic signal transduction, maintaining membrane integrity, fluidity, and ferroptosis [120]. The mevalonate pathway is a key pathway in cancer lipid metabolism and is primarily responsible for cholesterol synthesis. The initial step involves the condensation of acetyl-CoA with acetoacetyl-CoA to synthesize 3-hydroxy-3-methylglutaryl (HMG)-CoA, which is catalyzed by HMGCS. HMGCR, the rate-limiting enzyme in cholesterol biosynthesis, subsequently converts HMG-CoA to mevalonate. When sterol levels in endoplasmic reticulum (ER) membranes are sufficient, abundant HMGCR is rapidly degraded through ring finger protein 145 (RNF145) and glycoprotein 78 (gp78)-mediated ubiquitination. RNF145 is an endogenous sterol-responsive E3 ligase recruited to Insig proteins under sterol-sufficient conditions, which enhances the sterol-induced degradation of HMGCR, emphasizing the regulatory role of RNF145 in the mevalonate pathway and cholesterol synthesis [84]. Alternatively, a ubiquitin conjugating enzyme E2 G2 (UBE2G2)-dependent E3 ligase Hrd1 can regulate HMGCR activity in the absence of both RNF145 and gp78 [84]. Moreover, the membrane-bound RING-finger ubiquitin ligases gp78 and Trc8 can promote the ubiquitination and degradation of HMGCR in human fibroblasts [121]. Conversely, ubiquitin-specific protease 20 (USP20) has been identified as the feeding-responsive deubiquitinase of HMGCR [85]. Upon feeding-induced activation of mTORC1, USP20 undergoes phosphorylation, leading to its interaction with gp78 and subsequent stabilization of HMGCR. This mechanism highlights the intricate regulatory network involved in the posttranslational modification and turnover of HMGCR in response to nutritional cues [85]. HMGCR is physiologically activated as an unphosphorylated protein, while its phosphorylation at Ser872 attenuates HMGCR activity [122]. Previous studies have demonstrated that HMGCR can be phosphorylated in vitro by multiple protein kinases, including AMPK, PKC, and Ca^2+^ calmodulin-dependent kinase [82, 123]. In mouse hepatocytes and human HepG2 cells, thyroid-stimulating hormone (TSH) decreases the phosphorylation of HMGCR via inhibition of AMPK activity [82]. Both the biologically active form of dephosphorylated HMGCR and the HMGCR transcription are increased in the liver tissues of nonalcoholic fatty liver (NAFL) and nonalcoholic steatohepatitis (NASH) patients, suggesting severe cholesterol metabolism in patients with NAFLD [124]. Furthermore, miR-34a has been found to inhibit sirtuin-1 with downstream dephosphorylation of AMPK, resulting in reduced phosphorylation of HMGCR [83]. Given the association between NAFLD patients with obesity and dysregulated metabolic syndrome who are at high risk for nonviral HCC, it is plausible that there may be similarities in the phosphorylation and dephosphorylation of HMGCR between NAFLD and HCC patients. Additionally, further research is warranted to elucidate the roles of HMGCR ubiquitylation and phosphorylation in cancer cells.

## Targeting PTMs in reprogrammed lipid metabolism provides a therapeutic strategy for *cancer* treatment

Undoubtedly, abnormal lipid metabolism and signaling play a critical role in the metabolic transformation of cancer cells, enabling rapid proliferation, resistance to cell death, and metastasis in hostile environments. Considerable attention has been devoted to the development of antitumor drugs that target lipid metabolic reprogramming given their considerable clinical implications [2, 7]. Recent studies have indicated that targeting protein modifications in metabolic diseases and malignant cancer is an attractive field of study [125]. Innovative approaches, including adenoviral or lipid nanoparticle-based drug delivery and proteolysis-targeting chimeras (PROTACs), have demonstrated the feasibility of targeting protein modifications or protein modifiers to impede tumor progression in lung cancer [126]. Furthermore, kinase inhibitors targeting phosphorylation, antibodies against specific glycoforms targeting glycosylation, SUMO mimics targeting SUMOylation, CBP/p300 inhibitors targeting acetylation, and statins targeting lipidation have promising effects in early preclinical studies on malignant tumors, including prostate cancer [127]. Considering that SUMOylation is evidently enhanced in glioblastoma (GBM), novel natural and synthetic small-molecule inhibitors targeting SUMO-specific proteases (also known as SENPs), SAE1, and the SUMO-specific conjugating enzyme Ubc9 have demonstrated highly inhibitory effects on the SUMOylation of key proteins in GBM [128]. Given the posttranslational modifications involved in dysregulated lipid metabolism in cancer, we propose potential therapeutic strategies that target these posttranslational modifications (Table 2).

**Table 2:** Potential therapeutic strategies targeting PTMs in lipid metabolism

Protein	Drug	Targeting PTMs	Functions	Targets of inhibitors	Development stage	Refs
**ACLY**	Akt inhibitor VIII	Inhibits the phosphorylation of ACLY at Ser455	To reduce the intracellular lipid content and cell proliferation	AKT1, 2 and 3	In vitro and/or ex vivo use	[135]
**ACC**	Metformin	Promotes the phosphorylation of ACC	To inhibit lipid synthesis, and promote β-oxidation	Activates AMPK	Approved	[151, 152]
**ACC**	Berberine	Promotes the phosphorylation of ACC	To inhibit ACC and decrease intracellular fatty acid synthesis	Activates AMPK	Approved	[80]
**ACC**	Canagliflozin	Promotes the phosphorylation of ACC	To inhibit lipogenesis	Activates AMPK	Approved	[155]
**ACC**	Salicylate	Increases the phosphorylation of ACC	To suppress de novo lipogenesis, and inhibit the survival of prostate and lung cancer cells	Activates AMPK	Approved	[156]
**ACC**	MT 63–78	Promotes the phosphorylation of ACC	To suppress lipogenesis and the growth of prostate cancer cells	Activates AMPK	In vitro and/or ex vivo use	[157]
**SREBP-1c**	Berberine	Promotes the phosphorylation of SREBP-1c	To inhibit the cleavage and nuclear translocation of SREBP-1c in HCC cells and the liver of HFD-fad mice	Activates AMPK	Approved	[80]
	PhosTACs	Dephosphorylation	To facilitate the dephosphorylation of target	Recruits a Ser/Thr phosphatase to a phosphosubstrate to mediate its dephosphorylation	In vitro and/or ex vivo use	[160]
**PCAF, P300**	Isothiazolones (CCT077791 and CCT077792)		To suppress the cellular acetylation	To inhibit PCAF and P300	In vitro and/or ex vivo use	[168, 169]
**PCAF, P300**	Pyridoisothiazolones PU141		To induce cellular histone hypoacetylation and inhibit the proliferation of various neoplastic cells	To inhibit PCAF and P300	In vitro and/or ex vivo use	[168]
**P300**	C646		To suppress histone acetylation and radiosensitize NSCLC cells	To inhibit P300	In vitro and/or ex vivo use	[172]
**P300**	L002		To inhibit histone acetylation and suppress the growth of breast cancer cells	To inhibit P300	In vitro and/or ex vivo use	[173]
**PCAF, P300**	Garcinol derivative LTK-14		To suppress histone acetylation and induce the apoptosis of melanoma cells, and impair esophageal cancer metastasis	To inhibit PCAF and P300	In vitro and/or ex vivo use	[176, 177]
**P300, CBP**	Curcumin		To repress the acetylation of histone/nonhistone proteins	To inhibit P300/CBP	Phase III clinical trials for colorectal cancer	[204]
**PCAF**	Embelin		To suppress histone acetylation	To inhibit PCAF	In vitro and/or ex vivo use	[181]
**MDM2**	Nutlin-3		To suppress the growth of osteosarcoma and leukemia cells	To inhibit the interaction of MDM2-p53	Phase III clinical trials for leukemia	[205]
**MDM2**	DS-3032		To induce G1 cell cycle arrest, senescence and apoptosis	To inhibit MDM2	Phase 1 in AML	[206]
**MDM2**	CGM097		To inhibit the proliferation of tumor cells with wild type p53	To inhibit MDM2	Phase 1 in solid tumors	[207]
**MDM2**	APG-115		To induce tumor cell-cycle arrest and apoptosis in a p53-dependent manner	To inhibit the interaction of MDM2-p53	Phase 1/2 AML and CML	[208]
**USP14**	IU1		To suppress tumor cell growth	To inhibit USP14	In vitro and/or ex vivo use	[209]
**USP2**	ML364		To induce an increase in cellular cyclin D1 degradation and cause cell cycle arrest	To inhibit USP2 selectively	In vitro and/or ex vivo use	[192]
**HMGCR**	GSK2643943A	Promotes the ubiquitination of HMGCR	De-stabilizes HMGCR	To inhibit USP20	In vitro and/or ex vivo use	[85]
**HMGCR**	PROTAC P22A	Induces the ubiquitination and degradation of HMGCR	Degrades HMGCR	Recruits a specific ubiquitin ligase to substrate proteins for degradation	In vitro and/or ex vivo use	[197, 198]
**LXR**	PROTAC GW3965-PEG5-VH032 (3)	Induces the ubiquitination and degradation of LXR	Degrades LXR	Recruits a specific ubiquitin ligase to substrate proteins for degradation	In vitro and/or ex vivo use	[200]

### Kinase inhibitors targeting phosphorylation and PhosTACs to dephosphorylated target proteins

AKT, a key protein kinase in lipid metabolism, directly phosphorylates ACLY. AKT is widely recognized as a promising drug target for cancer therapy, and specific inhibitors of AKT exhibit favorable pharmaceutical properties. These AKT inhibitors can be categorized into ATP-competitive, allosteric, and irreversible inhibitors. ATP-competitive inhibitors (GSK690693, GDC0068, and AZD5363) [129–131], as well as allosteric inhibitors of AKT (MK-2206) [132–134], have been widely investigated in malignant tumor cells in vivo and in vitro. In pulmonary arterial vascular smooth muscle cells (PAVSMCs), the AKT inhibitor VIII effectively attenuates the activation of lipogenesis enzymes, particularly ACLY, by impairing the phosphorylation of ACLY at the Ser455 site. This inhibition results in reduced lipid accumulation and PAVSMC survival in pulmonary arterial hypertension [135]. Therefore, targeting ACLY phosphorylation by AKT inhibitors holds great promise for effectively disrupting lipid metabolism in cancer cells [135].

The mitogen-activated protein kinase (MAPK) pathway, which includes p38 MAPK, ERK, and JNK, plays a pivotal role in signal transduction in metabolic diseases and cancer [136, 137]. Accumulating evidence has demonstrated that ERK inhibitors are less susceptible to resistance mechanisms than inhibitors targeting upstream molecules in the MAPK pathway, such as RAF and MEK [138, 139]. In recent years, several ERK inhibitors that have shown encouraging results for cancer treatment have been introduced in clinical studies. They include SCH-772984, MK-8353, GDC-0994, ulixertinib (BVD-523), KO-947, and LY-3214996. Ulixertinib (BVD-523), an ATP-competitive kinase-selective inhibitor that targets phosphorylated ERK2 (pERK) and the downstream kinase RSK (pRSK), has demonstrated antitumor effects on NRAS-mutant melanoma and BRAF-mutant solid tumors [140]. Notably, ulixertinib has been found to be effective in several models showing intrinsic or acquired resistance to other MAPK pathway inhibitors [138]_._ Moreover, the combination of ERK inhibitors with upstream inhibitors exhibits synergistic benefits [141–143]. Selective inhibitors of JNK signaling, including SP600125, AS601245, JNK-IN-8, BI-78D3, XG-102, and the JNK inhibitor IX, have also exhibited excellent anticancer effects on solid and hematologic tumors [144, 145]. Notably, clinical studies on JNK inhibitors are currently inadequate, and increased attention should be directed toward this area. Additionally, p38 MAP kinase inhibitors have entered clinical trials for malignant tumor treatment. Ralimetinib, an extensively studied p38 MAPK inhibitor, has been shown to suppress tumor growth in various cancer models in vivo, including melanoma, non-small cell lung cancer, ovarian cancer, glioma, myeloma, and breast cancer models [146]. Talmapimod is another orally active, selective, and ATP-competitive p38α inhibitor that substantially abates human myeloma cell growth in vivo [147, 148]. However, research on whether MAPK inhibitors can directly impede abnormal lipid synthesis in cancer cells is limited. Evidence suggests that MAPKs play a direct role in controlling the activation and transcriptional activities of SREBP1 and SREBP2 by regulating the phosphorylation of SREBPs. Therefore, targeting the MAPK family members (p38 MAPK, ERK, and JNK) involved in lipid metabolism could represent a promising approach for cancer treatment.

AMPK contributes to fatty acid and cholesterol synthesis by phosphorylating key lipogenic enzymes and master transcription factors [17]. Numerous studies have suggested that therapies that activate AMPK are effective in treating various diseases, including cancer. Metformin, an indirect activator of AMPK and the first-line drug for the treatment of type II diabetes, has been reported to prevent the cleavage and activation of SREBP1 and SREBP2 [149]. Moreover, metformin increases the protein stability of Insig (a negative regulator of SREBPs), resulting in the decreased expression of lipogenic genes in mouse livers [150]. Additionally, in ovarian cancer cells, metformin attenuates lipid synthesis and enhances β-oxidation by augmenting the phosphorylation of ACC [151, 152]. Plant-derived products, such as BBR and quercetin, can also indirectly activate AMPK by impairing mitochondrial respiration. Notably, BBR promotes the phosphorylation of AMPK and SREBP-1c in HepG2 cells, leading to the inhibition of the cleavage and nuclear translocation of SREBP-1c [80]. BBR abates the phosphorylation and activity of ACC via the inactivation of AMPK in various cancer cells, including colon cancer, cervical carcinoma, and hepatocellular carcinoma cells [153, 154]. Canagliflozin, which is a sodium/glucose cotransporter inhibitor used to treat type 2 diabetes, has been recently found to suppress lipogenesis by promoting the AMPK-mediated phosphorylation of ACC in hepatocytes [155]. Direct AMPK activators containing salicylate, namely, MT 63–78 and 991, also inhibit proliferation and colony formation in multiple types of tumor cells by attenuating de novo lipogenesis [156, 157]. Bempedoic acid (ETC-1002), an AMPK activator and ACLY inhibitor, is currently in phase III clinical trials to reduce LDL-C in patients with hypercholesterolemia [158, 159]. Interestingly, in DEN/HFD-induced HCC models, the concurrent inhibition of ACLY and PD-L1 with bempedoic acid and anti-PD-L1 antibody remarkably suppresses hepatocarcinogenesis [55]. Overall, the activation of AMPK provides a hopeful therapeutic approach for cancer treatment by reducing de novo lipogenesis in cancer cells.

Considering adverse effects, such as off-target effects, toxicity, and drug resistance associated with kinase inhibition, the need to explore innovative strategies targeting protein phosphorylation is growing. Crews et al. has introduced a novel approach involving phosphorylation-targeting chimeras known as PhosTACs, which utilize PP2A to facilitate the dephosphorylation of target proteins [160]. PhosTACs represent potent tools for precisely modulating the function of target proteins, which employ heterobifunctional molecules to facilitate the formation of a complex between a phosphatase and specific phosphoprotein [161]. Given that it does not affect protein expression, potentially undesirable side effects caused by protein degradation can be avoided [160, 161]. Tau PhosTACs are applied in AD because tau PhosTACs can efficiently trigger the degradation of the tau protein by rapidly and sustainably inducing its dephosphorylation [162]. In addition, TKI-based PhosTAC can actively recruit phosphatases to remove phosphoric acid from EGFR while inhibiting the active center of the kinase, thus achieving the dual inhibition of EGFR [163]. Based on the evidence that the hyperphosphorylation of enzymes involved in lipid metabolism is a distinct feature in cancer cells, we propose PhosTACs as an encouraging new avenue for cancer treatment.

### Targeting acetylation by using small-molecule compound and natural inhibitors or inducing acetylation modification through AceTAG

Notably, targeting acetylation has emerged as a promising therapeutic strategy for treating metabolic diseases [164]. HDAC inhibitors and small-molecule inhibitors targeting histone acetyltransferases (HATs) are two well-established strategies that focus on the acetylation pathway in cancer therapy [165]. Small molecular inhibitors targeting HDAC (HDACi) play significant roles in cancer treatment through enhancing the efficacy of chemotherapy, inhibiting tumor growth and metastasis, regulating immune response, activating anti-inflammatory signaling pathways, and preventing drug resistance [166]. HDACi givinostat diminishes palmitic acid-induced intracellular lipid accumulation in HCC cell line HepG2 [167]. In a methionine-and choline-deficient diet (MCD) mouse model, hepatic inflammation and liver fibrosis are alleviated in the treatment with givinostat [167]. However, whether acetylation in cancer lipid metabolism could be altered by HDACi remains unclear. Moreover, PCAF and CBP/P300-mediated protein acetylation act as key drivers of dysregulated lipid metabolism in cancer cells. Small-molecule inhibitors, such as isothiazolones CCT077791 and CCT077792, have demonstrated markedly inhibitory effects on the activities of PCAF and P300 [168, 169]. The pyridisothiazolone PU141 has been shown to disrupt CBP and p300 selectively and inhibit the proliferation of various neoplastic cells in vivo and in vitro [170, 171]. C646, a selective and competitive inhibitor of p300, is one of the most widely studied inhibitors involving the modulation of p300-mediated histone modulation and signaling [172]. Furthermore, L002 has been identified as a potent, cell-permeable, reversible small molecule that inhibits p300 HAT activity in vitro. This compound effectively suppresses growth and prevents histone acetylation in in vivo and in vitro models of breast cancer cells [173]. Moreover, the natural product anacardic acid and its derivatives have been demonstrated to attenuate the HAT activity of p300 and PCAF [174, 175]. The natural product garcinol derivative LTK-14 inhibits p300 and PCAF [176, 177], which can induce the apoptosis of melanoma cells [178, 179] and inhibit esophageal cancer metastasis [177]. The natural phenolic compound curcumin, a novel p300/CREB-binding protein-specific inhibitor of acetyltransferase, has been found to reduce the expression of SREBP in vitro and to inhibit the target genes of SREBPs in liver or adipose tissues [180]. Additionally, embelin, a cell-permeable small molecule, has demonstrated a preferential inhibitory effect on PCAF compared with p300 and Tip60 HAT activity [181]. These synthetic molecules and natural inhibitors are extensively involved in the treatment of cancer cells by disturbing protein acetylation, indicating the potential applicability of targeting de novo lipogenesis.

AceTAG represents a pioneering acetylation tagging system that exploits endogenous CBP/p300 acetyltransferase to facilitate the acetylation of target proteins [182]. Notably, AceTAG efficiently induces the acetylation of histone H3.3, p65/RelA, and p53 in a rapid, selective, reversible, and controllable manner. This innovative approach holds great promise for modulating protein acetylation in cancer and lipid metabolism. Given that the acetylation of FASN promotes its degradation, the AceTAG system can be used to restrain de novo lipogenesis through inducing acetylation of FASN.

### Modulation of deubiquitinating enzyme (DUB) to decelerate lipid synthesis in *cancer* cells

Ubiquitination participates in modulating various cellular processes through proteolytic and nonproteolytic mechanisms. In this process, the evolutionarily conserved ubiquitin protein is attached to target proteins, where it exerts its regulatory effects [183]*.* This multistep process is carried out by ubiquitin-activating enzymes (E1), ubiquitin-conjugating enzymes (E2) and ubiquitin ligases (E3) [183]. Dysregulated ubiquitin signaling directly contributes to the initiation and progression of a broad spectrum of diseases, including malignant cancers [184]. As a result, ubiquitination enzymes have emerged as key targets for the development of anticancer drugs. Established strategies to inhibit ubiquitination-related factors include proteinase inhibitors, small-molecule inhibitors, or antagonists targeting E1/E2/E3 components [184]. Bortezomib, carfilzomib, and ixazomib are three protease inhibitors approved by the FDA and currently extensively utilized in the treatment of multiple myeloma [185]. A previous study has shown that bortezomib effectively reduces alcohol-induced lipogenesis and liver steatosis by downregulating the expression of SREBP-1c, FAS, and ACC in the livers of rats [186]. Furthermore, TAK-243 (MLN7243) is a specific inhibitor of ubiquitin-activating enzyme (UAE), which blocks ubiquitin conjugation and disrupts monoubiquitin signaling, as well as global protein ubiquitination, resulting in growth inhibition in solid and hematological tumors [187]. Within the realm of E3 ligases, MDM2 inhibitors such as Nutlin-3, DS-3032 (milademetan), CGM097, and APG-115, have emerged as innovative strategies for cancer therapy and shown potent antitumor effects [184, 188]. In the context of dysregulated lipid metabolism in cancer cells, the upregulation of deubiquitination often accelerates the growth of cancer cells in vivo and in vitro. Importantly, deubiquitinating enzymes (DUBs) are associated with all cancer hallmarks, making the modulation of their activity a promising therapeutic target [183]. IU1, the first highly selective inhibitor of USP14 that prevents its binding to the proteasome [189], might decrease lipid uptake and synthesis by interrupting the deubiquitination of USP14 on CD36 and FASN. Remarkably, IU1 effectively suppresses tumor growth in various cancer models [190, 191]. Additionally, USP2 enhances the protein stability of LDLR and FASN and has been identified as another promising target due to its oncogenic properties in vivo. The selective inhibitor of USP2, ML364, binds directly to USP2, leading to increased cyclin D1 degradation and subsequent cell cycle arrest [192, 193]. STD1T has been shown to inhibit USP2a enzymatic activity effectively in a concentration-dependent manner [194]. However, further research is required to determine whether small-molecule inhibitors of USP2 can impede de novo lipogenesis. One such example, USP20 interacts with gp78 and reduced the ubiquitination of HMGCR upon feeding-induced activation [85]. GSK2643943A, the specific inhibitor of USP20, can de-stabilize HMGCR and increase the ubiquitination and degradation of HMGCR [85].

### PROTACs as potential therapeutics for lipid metabolism

Remarkably, targeted protein degradation (TPD) technology, which includes proteolysis-targeting chimeras (PROTACs), molecular glues (MG), lysosome-targeting chimeras (LYTAC), chaperone-mediated autophagy (CMA)-targeting chimeras, autophagy-targeting chimera (AUTAC), autophagosome-tethering compound (ATTEC), and autophagy-targeting chimera (AUTOTAC), has emerged as a highly promising approach for eliminating specific disease-related proteins by harnessing cellular self-destruction mechanisms [195]. In this review, we emphasize the considerable potential of PROTACs, which recruit a specific ubiquitin ligase to substrate proteins for degradation and exhibit selectivity and availability for targeting PTMs involved in cancer lipid metabolism, offering a highly precise approach to protein modification [196]. P22A has been identified as a potent PROTAC molecule for triggering the substantial degradation of HMGCR that is located in the endoplasmic reticulum and is an essential enzyme for the conversion of mevalonate from HMG-CoA in lipid metabolism [197, 198]. The treatment of HCC cells with P22A causes a decrease in de novo cholesterol synthesis. Liver X receptors are members of the nuclear receptor family that promote cellular lipogenesis and maintain cholesterol homeostasis [199]. A recent study has designed a PROTAC (GW3965-PEG5-VH032) against LXR; this PROTAC effectively accelerates the degradation of the LXRβ protein in HuH-7 human hepatoma cells [200]. Given that the dysregulation of the PI3K–AKT signaling pathway contributes to metabolic reprogramming in various cancers, the selective degradation of PI3K using the PROTAC approach has been successfully designed, synthesized, and identified [201]. These compounds potently hinder the viability and proliferation of several cancer cells, including HCC, CRC, and cervical carcinoma cells [202, 203]. Overall, targeting the ubiquitination proteasome pathway by PROTACs has emerged as a promising strategy for achieving targeted degradation. We anticipate that this technology could be effectively utilized to develop agents against dysregulated lipid metabolism in cancer cells, with a particular focus on specific PTMs.

## Perspectives and conclusions

The advancement of mass spectrometry technology facilitated high-throughput, accurate, and sensitive measurement of PTM levels, enabling a deeper exploration of their functions, prevalence, and interactions. Li and colleagues analyzed the proteogenomic data combined with PTM profiles from 1,110 patients across 11 cancer types and revealed a common pattern of alterations in protein acetylation and phosphorylation that are intricately linked to the hallmarks of cancer [210]. Significantly, PTMs play a crucial role in the hallmark processes of cancer, particularly in the reprogramming of metabolism. Accumulating evidence indicated that targeting lipid metabolism could not only reduce cellular energy production but also boost the efficacy of chemotherapy and immunotherapy in diverse cancer models [2, 210, 211]. Notably, aberrant lipid metabolism is highly subject to PTMs in tumor cells, understanding the mechanisms underlying PTMs in highly detail can provide novel insight into therapeutic approaches. This review is dedicated to explore the PTMs in cancer lipid metabolism, with a primary focus on the phosphorylation, ubiquitylation, acetylation, SUMOylation, malonylation, and neddylation of key enzymes. We discuss diverse notable compounds that target either PTMs or upstream regulators in cancer lipid metabolism Recent studies have shown that mimicking the phosphorylation of ACC can effectively inhibit de novo lipogenesis and HCC and NSCLC progression. ACC inhibitors have been extensively researched for their ability to suppress ACC activity by modulating ACC phosphorylation. Prominent examples include the natural compound curcumin, along with small-molecule inhibitors such as MK-4074 [212], PF-05221304 [213], and firsocostat [214], which function by deactivating ACC through enhancing its phosphorylation and reducing its ubiquitination. Moreover, kinase inhibitors, including AKT and MAPK inhibitors, are promising approaches for targeting the phosphorylation of lipid metabolism enzymes and cancer treatment. Indirect AMPK activators, such as metformin and BBR, and the direct AMPK activators salicylate and MT 63–78 have shown notable inhibitory effects on de novo lipogenesis through promoting the phosphorylation of ACC and SREBPs. Additionally, the acetylation and deacetylation of key enzymes are promising targets in lipid metabolism. Small-molecule inhibitors and natural products that target PCAF and CBP/P300 are expected to block de novo lipogenesis by modulating the acetylation of lipid metabolism enzymes. Ubiquitylation is another important PTM that controls the protein activity and stability of metabolic enzymes and maintains intracellular lipid metabolic homeostasis. Protease inhibitors and DUB-selective inhibitors are widely used in modulating the ubiquitylation of lipid metabolism enzymes, thereby blocking lipogenesis in cancer cells.

PhosTAC, AceTAG, and PROTAC are heterobifunctional molecules that tether protein phosphatase, acetyltransferase or ubiquitin ligase to a protein of interest (POI), inducing the dephosphorylation, acetylation or ubiquitination of a POI, respectively [215]. Importantly, the development and application of these heterobifunctional molecules have provided novel tools and strategies for precise control over the modification status of proteins within cancer cells. These cutting-edge techniques offer practical avenues for regulating posttranslational modifications in lipid metabolism.

In summary, PTMs are dynamic mechanisms that regulate protein abundance, activity, function, subcellular localization, and interactions. PTMs are often involved in diseases related to lipid metabolism, offering unique and diverse mechanisms for regulating cancer cells. Therefore, gaining a deep understanding of PTM mechanisms may present an opportunity for innovative and refined drug development, leading to a precise and novel approach for the management of malignant tumors.

## Data Availability

No datasets were generated or analysed during the current study.

## Abbreviations

ACLY: ATP-citrate lyase
ACC: Acetyl-coA carboxylase
ACS: Acyl-coA synthetase
AMPK: AMP-activated protein kinase
ACS: Acyl-coA synthetase
AGPAT: Acylglycerolphosphate acyltransferase
AKT: Serine/threonine kinase 1
AceTAG: Acetylation-Targeting Chimera
BDK: Branched-chain alpha-keto acid dehydrogenase kinase
C/EBPα: CCAAT/enhancer-binding protein alpha
CPT: Carnitine palmitoyltransferase
CACT: Carnitine-acylcarnitine translocase
CSN6: Constitutive Photomorphogenesis 9 (COP9) signalosome (CSN) subunit 6
CDK8: Cyclin-dependent kinase 8
CRC: Colorectal cancer
DIO: Diet-induced obese
DGAT: Diacylglycerol acyltransferase
DDX39B: DExD-box helicase 39B
DUB: Deubiquitinating enzyme
ELOVL: Elongases of very long chain fatty acids
ERK: Extracellular signal-regulated kinase
EGFR: Epidermal growth factor receptor
FATPs: Fatty acid transport proteins
FABPs: Fatty acid-binding proteins
FASN: Fatty acid synthase
FAO: Fatty acid oxidation
FAT: Fatty acid translocase
FFAs: Free Fatty Acids
F6P: Fructose-6-phosphate
FBXW7: F-box and WD repeat domain containing 7
GPAT: Glycerol-3-phosphate acyltransferase
GSK3β: Glycogen synthase kinase-3β
HMGCR: 3-Hydroxy-3-methylglutaryl-CoA reductase
HSL: Hormone-sensitive lipase
HSP90β: Heat shock protein 90 beta
HRD1: HMG-CoA reductase degradation protein
HCC: Hepatocellular carcinoma
HDAC: Histone deacetylase
HAT: Histone acetyltransferases
IDOL: Inducible degrader of the low-density lipoprotein receptor
Insig: Insulin-inducible gene protein
JNK: C-Jun N-terminal kinases;
KLHL25: Kelch-like family member 25
KAT8: Lysine acetyltransferase 8
LDL: Low-density lipoprotein
LCFAs: Long-chain fatty acids
LDLR: Low-density lipoprotein receptor
LXR: Liver X receptor
mTORC1: Mechanistic/mammalian target of rapamycin complex 1
MUFA: Monounsaturated fatty acid
mTORC2: Mechanistic/mammalian target of rapamycin complex 2
MPC1: Mitochondrial pyruvate carrier 1
MAPK: Mitogen-activated protein kinase
NAFLD: Nonalcoholic fatty liver disease
NSCLC: Non-small cell lung cancer
NEDD8: Neural precursor cell expressed developmentally downregulated protein 8
PTM: Posttranslational modification
PAP: Phosphatidic acid phosphohydrolase
PKC: Protein kinase C
PKA: Protein kinase A
PATs: Palmitoyl acyltransferases
PI3K: Phosphoinositide 3-kinase
PCAF: P300/CBP-associated factor
PKM2: Pyruvate kinase M2
PROTAC: Proteolysis targeting chimera
PhosTAC: Phosphorylation targeting chimera
PD-L1: Programmed death-ligand 1
RNF145: Ring finger protein 145
SCD1: Stearoyl-coA desaturase 1
SREBP: Sterol regulatory element-binding protein
SIRT: Sirtuin; SNX8: Sorting nexin 8
Smurf1: Smad ubiquitylation regulatory factor-1
SUMO: Small ubiquitin-like modifier
TAG: Triacylglycerol
TCA: Tricarboxylic acid
TRIM21: Tripartite motif-containing 21
TRIM28: Tripartite motif-containing 28
USP: Ubiquitin-specific protease;
UBE2M: Ubiquitin conjugating enzyme E2 M
UBE2G2: Ubiquitin conjugating enzyme E2 G2
VLDL: Very-low-density lipoprotein
